# 2,2,2-Tribromo-*N*-(3-methyl­phen­yl)acetamide

**DOI:** 10.1107/S160053680905048X

**Published:** 2009-11-28

**Authors:** B. Thimme Gowda, Sabine Foro, P. A. Suchetan, Hartmut Fuess

**Affiliations:** a Department of Chemistry, Mangalore University, Mangalagangotri 574 199, Mangalore, India; b Institute of Materials Science, Darmstadt University of Technology, Petersenstrasse 23, D-64287 Darmstadt, Germany

## Abstract

The asymmetric unit of the title compound, C_9_H_8_Br_3_NO, contains two independent mol­ecules. The conformation of the N—H bond is *anti* to the 3-methyl substituent in the benzene ring in each mol­ecule. The structure shows both intra­molecular N—H⋯Br and inter­molecular N—H⋯O hydrogen bonding, the latter leading to the formation of helical supra­molecular chains along the *b* axis.

## Related literature

For preparation of the compound, see: Gowda *et al.* (2003 ▶). For our study of the effect of ring and side-chain substituents on the solid-state structures of *N*-aromatic amides, see: Gowda *et al.* (2007**a* ▶,b* ▶, 2009 ▶). For the structures of other amides, see: Brown (1966 ▶).
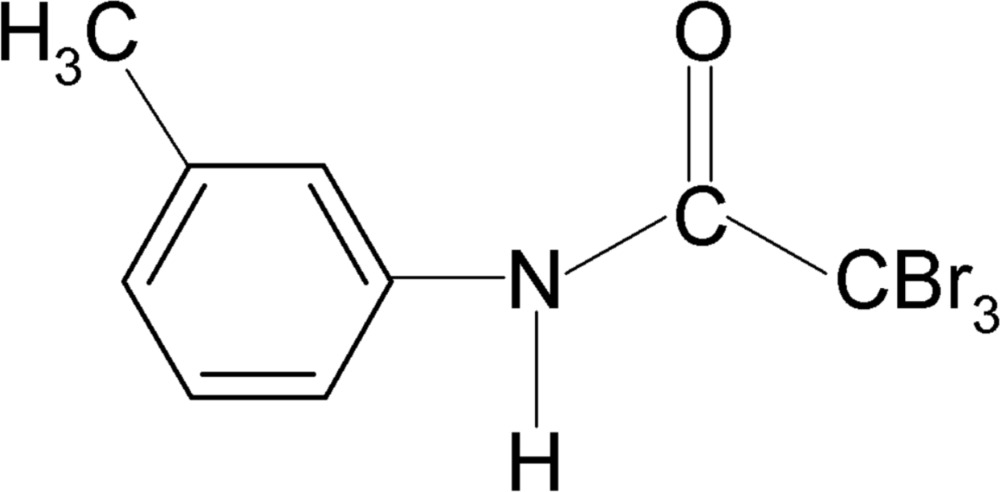


## Experimental

### 

#### Crystal data

C_9_H_8_Br_3_NO
*M* *_r_* = 385.89
Monoclinic, 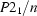

*a* = 11.360 (1) Å
*b* = 10.280 (1) Å
*c* = 20.298 (3) Å
β = 100.23 (1)°
*V* = 2332.7 (5) Å^3^
*Z* = 8
Cu *K*α radiation
μ = 12.58 mm^−1^
*T* = 299 K
0.28 × 0.13 × 0.08 mm

#### Data collection

Enraf–Nonius CAD-4 diffractometer
Absorption correction: ψ scan (North *et al.*, 1968 ▶) *T*_min_ = 0.127, *T*_max_ = 0.433
4358 measured reflections
4147 independent reflections
2939 reflections with *I* > 2σ(*I*)
*R*_int_ = 0.059

#### Refinement

*R*[*F* ^2^ > 2σ(*F* ^2^)] = 0.080
*wR*(*F* ^2^) = 0.249
*S* = 1.04
4147 reflections
255 parameters
H-atom parameters constrained
Δρ_max_ = 1.60 e Å^−3^
Δρ_min_ = −1.66 e Å^−3^

### 

Data collection: *CAD-4-PC* (Enraf–Nonius, 1996 ▶); cell refinement: *CAD-4-PC*; data reduction: *REDU4* (Stoe & Cie, 1987 ▶); program(s) used to solve structure: *SHELXS97* (Sheldrick, 2008 ▶); program(s) used to refine structure: *SHELXL97* (Sheldrick, 2008 ▶); molecular graphics: *PLATON* (Spek, 2009 ▶); software used to prepare material for publication: *SHELXL97*.

## Supplementary Material

Crystal structure: contains datablocks I, global. DOI: 10.1107/S160053680905048X/tk2583sup1.cif

Structure factors: contains datablocks I. DOI: 10.1107/S160053680905048X/tk2583Isup2.hkl

Additional supplementary materials:  crystallographic information; 3D view; checkCIF report

## Figures and Tables

**Table 1:** Hydrogen-bond geometry (Å, °)

*D*—H⋯*A*	*D*—H	H⋯*A*	*D*⋯*A*	*D*—H⋯*A*
N1—H1*N*⋯O2^i^	0.86	2.21	3.005 (11)	153
N1—H1*N*⋯Br1	0.86	2.60	3.068 (8)	115
N2—H2*N*⋯O1^ii^	0.86	2.11	2.886 (11)	150
N2—H2*N*⋯Br6	0.86	2.61	3.100 (8)	117

Symmetry codes: (i) 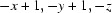; (ii) 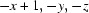.

**Fig. 1.**
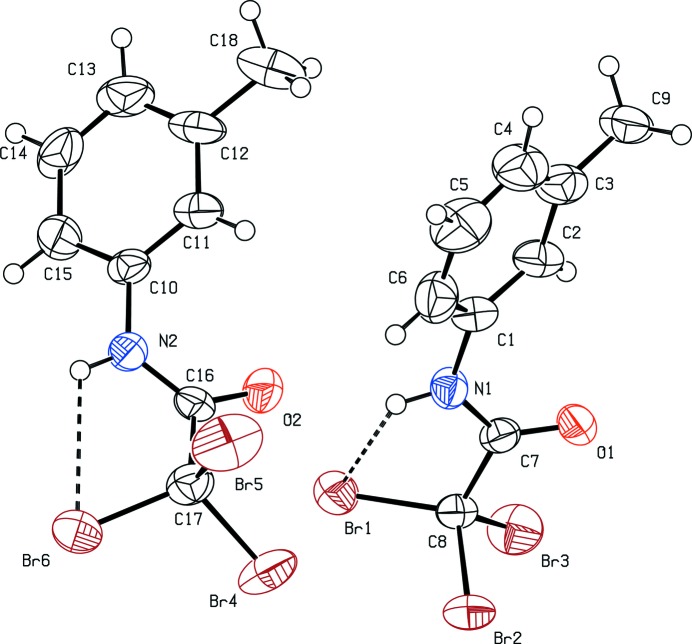
Molecular structures of the two independent molecules of (I), showing the atom labelling scheme. Displacement ellipsoids are drawn at the 50% probability level and H atoms are represented as small spheres of arbitrary radii.

**Fig. 2.**
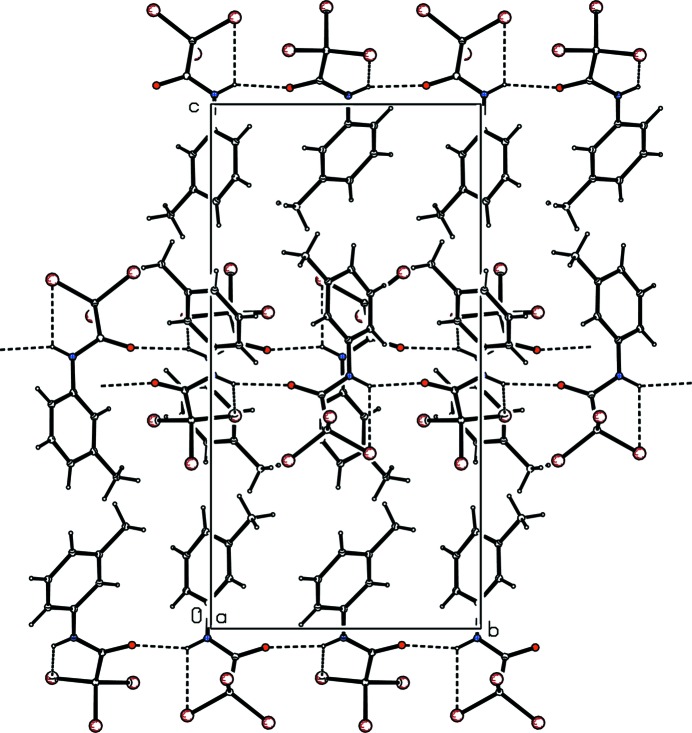
Molecular packing in (I) with hydrogen bonds shown as dashed lines.

## supplementary crystallographic information

### Comment

As part of a study of the effect of the ring and the side-chain substituents on the solid-state structures of *N*-aromatic amides (Gowda *et al.*, 2007*a, b*, 2009), in the present work, the structure of *N*-(3-methylphenyl)2,2,2-tribromoacetamide (I) has been determined (Fig. 1). The asymmetric unit of the structure contains two independent molecules. The conformation of the N—H bond is *anti* to the 3-methyl substituent in the benzene ring in each molecule, similar to that observed in *N*-(3-methylphenyl)2,2,2-trichloroacetamide (Gowda *et al.*, 2007*a*) and *N*-(3-methylphenyl)2,2,2-trimethylacetamide (Gowda *et al.*, 2007*b*). Further, the conformation of the N—H bond in the structure is *anti* to the C═O bond in the side-chain, similar to that observed in *N*-(phenyl)2,2,2-tribromoacetamide (Gowda *et al.*, 2009) and other amides (Brown, 1966; Gowda *et al.*, 2007*a,b*). The structure of (I) shows both the intramolecular N—H···Br and intermolecular N—H···O hydrogen bonding, Table 1. The packing diagram showing the hydrogen bonds (Table 1) and the supramolecular chains parallel to the *b* axis is shown in Fig. 2.

### Experimental

The title compound was prepared from *m*-toluidine, tribromoacetic acid and phosphorylchloride according to the literature method (Gowda *et al.*, 2003). Single crystals of (I) were obtained by the slow evaporation of its petroleum ether solution at room temperature.

### Refinement

The H atoms were positioned with idealized geometry using a riding model with N—H = 0.86 Å and C—H = 0.93–0.96 Å, and with *U*_iso_ = 1.2*U*_eq_(carrier atom).

The residual electron-density features are located in the regions of Br2 and Br1. The highest peak was 0.84 Å from Br2 and the deepest hole was 0.99 Å from Br1.

### Crystal data

**Table:**

C_9_H_8_Br_3_NO	*F*(000) = 1456
*M**_r_* = 385.89	*D*_x_ = 2.198 Mg m^−^^3^
Monoclinic, *P*2_1_/*n*	Cu *K*α radiation, λ = 1.54180 Å
Hall symbol: -P 2yn	Cell parameters from 25 reflections
*a* = 11.360 (1) Å	θ = 4.9–20.6°
*b* = 10.280 (1) Å	µ = 12.58 mm^−^^1^
*c* = 20.298 (3) Å	*T* = 299 K
β = 100.23 (1)°	Rod, colourless
*V* = 2332.7 (5) Å^3^	0.28 × 0.13 × 0.08 mm
*Z* = 8	

### Data collection

**Table:**

Enraf–Nonius CAD-4 diffractometer	2939 reflections with *I* > 2σ(*I*)
Radiation source: fine-focus sealed tube	*R*_int_ = 0.059
graphite	θ_max_ = 67.0°, θ_min_ = 4.2°
ω/2θ scans	*h* = −13→1
Absorption correction: ψ scan (North *et al.*, 1968)	*k* = −12→0
*T*_min_ = 0.127, *T*_max_ = 0.433	*l* = −23→24
4358 measured reflections	3 standard reflections every 120 min
4147 independent reflections	intensity decay: 1.0%

### Refinement

**Table:**

Refinement on *F*^2^	Primary atom site location: structure-invariant direct methods
Least-squares matrix: full	Secondary atom site location: difference Fourier map
*R*[*F*^2^ > 2σ(*F*^2^)] = 0.080	Hydrogen site location: inferred from neighbouring sites
*wR*(*F*^2^) = 0.249	H-atom parameters constrained
*S* = 1.04	*w* = 1/[σ^2^(*F*_o_^2^) + (0.1741*P*)^2^ + 2.28*P*] where *P* = (*F*_o_^2^ + 2*F*_c_^2^)/3
4147 reflections	(Δ/σ)_max_ < 0.001
255 parameters	Δρ_max_ = 1.60 e Å^−^^3^
0 restraints	Δρ_min_ = −1.66 e Å^−^^3^

### Special details

**Table:**

Geometry. All e.s.d.'s (except the e.s.d. in the dihedral angle between two l.s. planes) are estimated using the full covariance matrix. The cell e.s.d.'s are taken into account individually in the estimation of e.s.d.'s in distances, angles and torsion angles; correlations between e.s.d.'s in cell parameters are only used when they are defined by crystal symmetry. An approximate (isotropic) treatment of cell e.s.d.'s is used for estimating e.s.d.'s involving l.s. planes.
Refinement. Refinement of *F*^2^ against ALL reflections. The weighted *R*-factor *wR* and goodness of fit *S* are based on *F*^2^, conventional *R*-factors *R* are based on *F*, with *F* set to zero for negative *F*^2^. The threshold expression of *F*^2^ > σ(*F*^2^) is used only for calculating *R*-factors(gt) *etc*. and is not relevant to the choice of reflections for refinement. *R*-factors based on *F*^2^ are statistically about twice as large as those based on *F*, and *R*- factors based on ALL data will be even larger.

### Fractional atomic coordinates and isotropic or equivalent isotropic displacement parameters (Å^2^)

**Table:**

	*x*	*y*	*z*	*U*_iso_*/*U*_eq_	
C1	0.6706 (9)	0.5084 (11)	−0.0373 (5)	0.042 (2)	
C2	0.6595 (10)	0.4132 (11)	−0.0870 (5)	0.049 (3)	
H2	0.7156	0.3465	−0.0832	0.059*	
C3	0.5676 (11)	0.4153 (13)	−0.1414 (6)	0.059 (3)	
C4	0.4854 (11)	0.5134 (15)	−0.1451 (7)	0.068 (3)	
H4	0.4246	0.5196	−0.1822	0.082*	
C5	0.4922 (13)	0.6036 (15)	−0.0939 (8)	0.078 (4)	
H5	0.4324	0.6660	−0.0959	0.094*	
C6	0.5834 (11)	0.6036 (12)	−0.0409 (7)	0.060 (3)	
H6	0.5874	0.6663	−0.0076	0.072*	
C7	0.8238 (9)	0.4032 (9)	0.0454 (5)	0.038 (2)	
C8	0.9316 (9)	0.4273 (9)	0.1025 (5)	0.041 (2)	
C9	0.5614 (13)	0.3160 (14)	−0.1948 (6)	0.068 (4)	
H9A	0.5043	0.2503	−0.1886	0.082*	
H9B	0.6387	0.2768	−0.1928	0.082*	
H9C	0.5373	0.3565	−0.2377	0.082*	
Br1	1.01398 (11)	0.58885 (12)	0.09396 (6)	0.0534 (4)	
Br2	0.86902 (12)	0.42753 (12)	0.18531 (5)	0.0544 (4)	
Br3	1.04582 (12)	0.28886 (14)	0.10312 (7)	0.0645 (4)	
N1	0.7674 (7)	0.5085 (8)	0.0170 (4)	0.0396 (18)	
H1N	0.7923	0.5829	0.0332	0.047*	
O1	0.7944 (8)	0.2926 (7)	0.0314 (4)	0.055 (2)	
C10	0.1952 (9)	−0.0100 (10)	0.0500 (5)	0.041 (2)	
C11	0.2737 (10)	0.0681 (10)	0.0959 (5)	0.047 (2)	
H11	0.3299	0.1209	0.0808	0.056*	
C12	0.2665 (13)	0.0654 (12)	0.1619 (5)	0.060 (3)	
C13	0.1874 (14)	−0.0199 (13)	0.1849 (6)	0.070 (4)	
H13	0.1880	−0.0289	0.2305	0.084*	
C14	0.1082 (14)	−0.0910 (12)	0.1392 (7)	0.068 (4)	
H14	0.0501	−0.1408	0.1543	0.082*	
C15	0.1128 (12)	−0.0905 (11)	0.0720 (6)	0.056 (3)	
H15	0.0619	−0.1428	0.0422	0.067*	
C16	0.2210 (9)	0.0901 (9)	−0.0539 (5)	0.041 (2)	
C17	0.2661 (11)	0.0687 (11)	−0.1209 (6)	0.051 (3)	
C18	0.3472 (14)	0.1501 (18)	0.2107 (7)	0.084 (5)	
H18A	0.3064	0.2295	0.2174	0.101*	
H18B	0.4184	0.1696	0.1933	0.101*	
H18C	0.3682	0.1052	0.2526	0.101*	
Br4	0.22259 (14)	0.21315 (14)	−0.17949 (7)	0.0697 (5)	
Br5	0.44004 (14)	0.05889 (19)	−0.09624 (9)	0.0877 (6)	
Br6	0.21012 (18)	−0.08796 (14)	−0.16568 (7)	0.0792 (5)	
N2	0.2067 (9)	−0.0130 (8)	−0.0181 (4)	0.047 (2)	
H2N	0.2041	−0.0876	−0.0375	0.056*	
O2	0.2111 (8)	0.2020 (7)	−0.0359 (4)	0.0539 (19)	

### Atomic displacement parameters (Å^2^)

**Table:**

	*U*^11^	*U*^22^	*U*^33^	*U*^12^	*U*^13^	*U*^23^
C1	0.043 (5)	0.053 (6)	0.031 (5)	0.005 (5)	0.007 (4)	0.005 (4)
C2	0.041 (5)	0.068 (7)	0.035 (5)	0.000 (5)	0.002 (4)	0.003 (5)
C3	0.052 (6)	0.083 (9)	0.041 (6)	−0.015 (6)	0.009 (5)	0.008 (6)
C4	0.051 (7)	0.089 (10)	0.059 (8)	−0.008 (7)	−0.006 (6)	0.007 (7)
C5	0.064 (8)	0.084 (10)	0.083 (11)	0.020 (8)	0.000 (7)	0.020 (8)
C6	0.056 (7)	0.052 (7)	0.070 (8)	−0.010 (5)	0.007 (6)	0.005 (6)
C7	0.046 (5)	0.037 (5)	0.031 (5)	0.005 (4)	0.007 (4)	0.004 (4)
C8	0.048 (6)	0.043 (5)	0.030 (5)	0.001 (4)	0.003 (4)	0.002 (4)
C9	0.083 (9)	0.087 (9)	0.033 (6)	−0.020 (7)	0.007 (6)	−0.004 (6)
Br1	0.0538 (7)	0.0568 (7)	0.0478 (7)	−0.0096 (5)	0.0043 (5)	−0.0019 (5)
Br2	0.0668 (8)	0.0680 (8)	0.0295 (6)	0.0008 (6)	0.0110 (5)	0.0026 (5)
Br3	0.0608 (8)	0.0645 (8)	0.0660 (8)	0.0231 (6)	0.0057 (6)	0.0021 (6)
N1	0.036 (4)	0.035 (4)	0.045 (5)	−0.001 (3)	0.000 (3)	0.002 (3)
O1	0.078 (5)	0.037 (4)	0.043 (4)	−0.003 (4)	−0.009 (4)	−0.003 (3)
C10	0.057 (6)	0.036 (5)	0.031 (5)	0.013 (4)	0.010 (4)	0.003 (4)
C11	0.055 (6)	0.052 (6)	0.033 (5)	0.014 (5)	0.005 (4)	0.002 (4)
C12	0.080 (8)	0.070 (8)	0.026 (5)	0.020 (6)	−0.004 (5)	−0.004 (5)
C13	0.110 (11)	0.067 (8)	0.037 (6)	0.019 (8)	0.029 (7)	−0.001 (6)
C14	0.108 (11)	0.050 (7)	0.059 (8)	−0.007 (7)	0.051 (8)	0.004 (5)
C15	0.077 (8)	0.048 (6)	0.046 (6)	−0.003 (6)	0.024 (6)	−0.010 (5)
C16	0.052 (6)	0.042 (5)	0.028 (5)	0.000 (4)	0.006 (4)	−0.007 (4)
C17	0.058 (7)	0.055 (6)	0.043 (6)	0.001 (5)	0.016 (5)	0.005 (5)
C18	0.086 (10)	0.118 (13)	0.042 (7)	0.010 (9)	−0.007 (7)	−0.016 (8)
Br4	0.0915 (10)	0.0705 (9)	0.0531 (8)	0.0183 (7)	0.0289 (7)	0.0263 (6)
Br5	0.0563 (8)	0.1225 (15)	0.0868 (12)	0.0133 (8)	0.0192 (8)	0.0250 (10)
Br6	0.1327 (15)	0.0674 (9)	0.0405 (7)	−0.0189 (8)	0.0231 (8)	−0.0114 (6)
N2	0.072 (6)	0.035 (4)	0.035 (4)	−0.001 (4)	0.015 (4)	−0.003 (3)
O2	0.079 (5)	0.033 (4)	0.050 (4)	0.003 (4)	0.013 (4)	0.002 (3)

### Geometric parameters (Å, °)

**Table:**

C1—C6	1.385 (16)	C10—C15	1.382 (16)
C1—C2	1.394 (15)	C10—N2	1.411 (12)
C1—N1	1.412 (12)	C10—C11	1.419 (15)
C2—C3	1.379 (16)	C11—C12	1.357 (15)
C2—H2	0.9300	C11—H11	0.9300
C3—C4	1.367 (19)	C12—C13	1.39 (2)
C3—C9	1.482 (17)	C12—C18	1.503 (18)
C4—C5	1.38 (2)	C13—C14	1.38 (2)
C4—H4	0.9300	C13—H13	0.9300
C5—C6	1.354 (18)	C14—C15	1.373 (16)
C5—H5	0.9300	C14—H14	0.9300
C6—H6	0.9300	C15—H15	0.9300
C7—O1	1.205 (12)	C16—O2	1.218 (12)
C7—N1	1.336 (12)	C16—N2	1.311 (13)
C7—C8	1.548 (14)	C16—C17	1.553 (14)
C8—Br3	1.925 (10)	C17—Br6	1.902 (12)
C8—Br1	1.929 (10)	C17—Br4	1.912 (11)
C8—Br2	1.938 (10)	C17—Br5	1.953 (12)
C9—H9A	0.9600	C18—H18A	0.9600
C9—H9B	0.9600	C18—H18B	0.9600
C9—H9C	0.9600	C18—H18C	0.9600
N1—H1N	0.8600	N2—H2N	0.8600
			
C6—C1—C2	119.0 (10)	C15—C10—N2	119.4 (9)
C6—C1—N1	119.4 (10)	C15—C10—C11	120.6 (10)
C2—C1—N1	121.5 (9)	N2—C10—C11	119.9 (10)
C3—C2—C1	121.8 (11)	C12—C11—C10	119.7 (12)
C3—C2—H2	119.1	C12—C11—H11	120.1
C1—C2—H2	119.1	C10—C11—H11	120.1
C4—C3—C2	117.9 (12)	C11—C12—C13	119.9 (12)
C4—C3—C9	121.7 (12)	C11—C12—C18	120.2 (14)
C2—C3—C9	120.4 (12)	C13—C12—C18	119.8 (12)
C3—C4—C5	120.5 (12)	C14—C13—C12	119.3 (11)
C3—C4—H4	119.8	C14—C13—H13	120.3
C5—C4—H4	119.8	C12—C13—H13	120.3
C6—C5—C4	121.9 (13)	C15—C14—C13	122.0 (12)
C6—C5—H5	119.1	C15—C14—H14	119.0
C4—C5—H5	119.1	C13—C14—H14	119.0
C5—C6—C1	118.8 (13)	C14—C15—C10	118.1 (11)
C5—C6—H6	120.6	C14—C15—H15	120.9
C1—C6—H6	120.6	C10—C15—H15	120.9
O1—C7—N1	124.8 (9)	O2—C16—N2	124.8 (9)
O1—C7—C8	118.5 (8)	O2—C16—C17	117.3 (9)
N1—C7—C8	116.6 (8)	N2—C16—C17	117.6 (9)
C7—C8—Br3	109.2 (6)	C16—C17—Br6	113.8 (7)
C7—C8—Br1	113.8 (6)	C16—C17—Br4	110.2 (7)
Br3—C8—Br1	107.4 (5)	Br6—C17—Br4	109.5 (6)
C7—C8—Br2	106.6 (7)	C16—C17—Br5	105.0 (7)
Br3—C8—Br2	110.2 (5)	Br6—C17—Br5	108.4 (6)
Br1—C8—Br2	109.6 (5)	Br4—C17—Br5	109.7 (6)
C3—C9—H9A	109.5	C12—C18—H18A	109.5
C3—C9—H9B	109.5	C12—C18—H18B	109.5
H9A—C9—H9B	109.5	H18A—C18—H18B	109.5
C3—C9—H9C	109.5	C12—C18—H18C	109.5
H9A—C9—H9C	109.5	H18A—C18—H18C	109.5
H9B—C9—H9C	109.5	H18B—C18—H18C	109.5
C7—N1—C1	125.7 (9)	C16—N2—C10	124.5 (8)
C7—N1—H1N	117.1	C16—N2—H2N	117.7
C1—N1—H1N	117.1	C10—N2—H2N	117.7
			
C6—C1—C2—C3	3.6 (17)	C15—C10—C11—C12	−1.1 (16)
N1—C1—C2—C3	−177.6 (10)	N2—C10—C11—C12	−176.5 (10)
C1—C2—C3—C4	−1.0 (17)	C10—C11—C12—C13	4.1 (17)
C1—C2—C3—C9	177.3 (10)	C10—C11—C12—C18	−178.5 (11)
C2—C3—C4—C5	−3(2)	C11—C12—C13—C14	−7(2)
C9—C3—C4—C5	178.9 (12)	C18—C12—C13—C14	175.8 (13)
C3—C4—C5—C6	4(2)	C12—C13—C14—C15	7(2)
C4—C5—C6—C1	−2(2)	C13—C14—C15—C10	−4(2)
C2—C1—C6—C5	−2.3 (18)	N2—C10—C15—C14	176.3 (11)
N1—C1—C6—C5	178.9 (11)	C11—C10—C15—C14	0.9 (17)
O1—C7—C8—Br3	33.1 (12)	O2—C16—C17—Br6	−151.8 (9)
N1—C7—C8—Br3	−149.3 (7)	N2—C16—C17—Br6	34.1 (13)
O1—C7—C8—Br1	153.1 (8)	O2—C16—C17—Br4	−28.4 (13)
N1—C7—C8—Br1	−29.2 (11)	N2—C16—C17—Br4	157.5 (8)
O1—C7—C8—Br2	−86.0 (10)	O2—C16—C17—Br5	89.7 (10)
N1—C7—C8—Br2	91.6 (9)	N2—C16—C17—Br5	−84.4 (10)
O1—C7—N1—C1	−5.2 (17)	O2—C16—N2—C10	−9.8 (18)
C8—C7—N1—C1	177.3 (9)	C17—C16—N2—C10	163.8 (10)
C6—C1—N1—C7	146.8 (11)	C15—C10—N2—C16	139.2 (11)
C2—C1—N1—C7	−32.0 (15)	C11—C10—N2—C16	−45.4 (15)

### Hydrogen-bond geometry (Å, °)

**Table:**

*D*—H···*A*	*D*—H	H···*A*	*D*···*A*	*D*—H···*A*
N1—H1N···O2^i^	0.86	2.21	3.005 (11)	153
N1—H1N···Br1	0.86	2.60	3.068 (8)	115
N2—H2N···O1^ii^	0.86	2.11	2.886 (11)	150
N2—H2N···Br6	0.86	2.61	3.100 (8)	117

Symmetry codes: (i) −*x*+1, −*y*+1, −*z*; (ii) −*x*+1, −*y*, −*z*.
